# Can a Single Representational Object Account for Different Number-Space Mappings?

**DOI:** 10.3389/fnhum.2021.750964

**Published:** 2021-10-04

**Authors:** Arnaud Viarouge, Maria Dolores de Hevia

**Affiliations:** ^1^Université de Paris, LaPsyDÉ, CNRS, Paris, France; ^2^Université de Paris, INCC UMR 8002, CNRS, Paris, France

**Keywords:** number, space, mappings, SCE, spatial–numerical association of response code (SNARC)

## Abstract

Numbers are mapped onto space from birth on, as evidenced by a variety of interactions between the processing of numerical and spatial information. In particular, larger numbers are associated to larger spatial extents (number/spatial extent mapping) and to rightward spatial locations (number/location mapping), and smaller numbers are associated to smaller spatial extents and leftward spatial locations. These two main types of number/space mappings (number/spatial extent and number/location mappings) are usually assumed to reflect the fact that numbers are represented on an internal continuum: the mental number line. However, to date there is very little evidence that these two mappings actually reflect a single representational object. Across two experiments in adults, we investigated the interaction between number/location and number/spatial extent congruency effects, both when numbers were presented in a non-symbolic and in a symbolic format. We observed a significant interaction between the two mappings, but only in the context of an implicit numerical task. The results were unaffected by the format of presentation of numbers. We conclude that the number/location and the number/spatial extent mappings can stem from the activation of a single representational object, but only in specific experimental contexts.

## Introduction

Humans possess an inborn ability to represent, discriminate, and manipulate numerical quantities, an ability that is shared with many other species (Cantlon, 2018). This ability is supported by the approximate number system (or ANS, Burr and Ross, 2008; Dehaene, 2009; Odic and Starr, 2018), which allows us to estimate (and mentally manipulate) the approximate numerosity of a set without using any symbolic knowledge (language or counting). The main signature of this cognitive system is that the variable determining a successful discrimination is the ratio between the two numerosities to compare, so that the larger the ratio between them the better the discrimination, a signature that also governs discrimination for other continuous, perceptual dimensions (Feigenson et al., 2004). This core, numerical cognitive system is thought to show continuity in development, and therefore to support the acquisition of formal math and symbolic numerical representations, with individual differences in numerical acuity predicting, and correlating with, math scores later on in life (Halberda et al., 2008; Starr et al., 2013). Another crucial aspect characterizing numerical representations is their spatial signature. In fact, a widely accepted view on numerical cognition considers numbers as distributions of activation along a spatially oriented mental number line (Restle, 1970; Dehaene, 1992).

Interactions between number and spatial representations have been extensively described in the literature, using a variety of methods: from visuo-spatial tasks, such as line bisection and reproduction tasks, where numbers impact spatial performance (Fischer, 2001; de Hevia et al., 2006, 2008; de Hevia and Spelke, 2009; Viarouge and de Hevia, 2013), to numerical tasks, such as parity judgment or magnitude comparison, where visuo-spatial variables modulate numerical judgments (Dehaene et al., 1993; Fias, 1996; Fischer et al., 2003; Bulf et al., 2014). This bidirectional interaction has been described across ages in humans, from infants who from birth spontaneously associate small numbers to small spatial extents and leftward spatial locations and large numbers to large spatial extents and rightward spatial locations (de Hevia and Spelke, 2010; de Hevia et al., 2014, 2017; Bulf et al., 2016; Di Giorgio et al., 2019), through childhood (Girelli et al., 2000; de Hevia and Spelke, 2009; Patro and Haman, 2012) and up to adulthood (Dehaene et al., 1993; Fischer et al., 2003), and even in non-human animals, such as chicks and chimpanzees, who have been shown to exhibit lateralized spatial associations of numerical quantities similar to humans (Adachi, 2014; Drucker and Brannon, 2014; Rugani et al., 2015).

In general terms, the interactions between numerical and spatial information can be described according to two types of number-space mappings (de Hevia, 2021). On the one hand, numbers can be mapped onto corresponding spatial extents, with larger numbers associated with larger spatial extents. This type of mapping is well exemplified in the Stroop paradigm, also known as the size congruity effect (SCE): when subjects have to compare numerical quantities (be it in a symbolic or non-symbolic format), the information of physical size interacts with the quantity judgment, so that incongruent combinations (e.g., a small quantity occupying a large space) yield worse performance than congruent combinations (e.g., a small quantity occupying a small space). This type of interaction between size and number has also been described in line bisection tasks (e.g., de Hevia et al., 2006; de Hevia and Spelke, 2009) as well as in reproduction tasks (e.g., de Hevia et al., 2008; Viarouge and de Hevia, 2013), providing further support to the idea that number is mapped onto a corresponding physical spatial extent.

On the other hand, numbers can be associated with different, lateralized spatial locations. This phenomenon is represented by the Spatial Numerical Association of Response Codes (SNARC) effect, by which small numbers are associated with the left and large numbers with the right side of space (Dehaene et al., 1993), obtaining an advantage in performance when the response side/or number location and the numerical magnitude are aligned according to a left-to-right oriented representation, with increasing numbers toward the right. This effect might be modulated by factors such as scanning habits (left-to-right vs. right-to-left writing/reading direction: Shaki et al., 2009), and contextual experience factors (Pitt and Casasanto, 2020).

Besides a few exceptions (de Hevia et al., 2006, 2008; Cipora et al., 2020; de Hevia, 2021), most authors usually assume that these two types of mappings (number-extent and number-location) reflect the same representational object, often appealing to the activation of a “mental number line” when interpreting the source of the interaction between numerical and spatial information. In fact, any type of behavioral interaction between numerical and spatial information in a given task is accounted for by the idea that humans might represent different numerosities along an internal spatial continuum: the mental number line.

However, some findings cast doubt on this generally accepted assumption. First, humans at birth show evidence of a dissociation between the number-spatial extent and the number-spatial location mappings. In particular, from birth and during the first year of life, infants spontaneously create mappings between number and spatial extent that can be generalized to the dimension of time (de Hevia and Spelke, 2010; Srinivasan and Carey, 2010; de Hevia et al., 2014). In some conditions, newborns and infants are also able to create mappings between number and brightness (de Hevia and Spelke, 2013; Bonn et al., 2019) and brightness and loudness (Lewkowicz and Turkewitz, 1980), supporting the view that number-spatial extent mapping is not specific to numerical information and extends to other quantitative dimensions. However, while at birth and during infancy humans associate lateralized spatial locations (left vs. right) to different numerosities (small vs. large, respectively), this number-spatial location mapping does not generalize to dimensions other than number, like size and brightness (Bulf et al., 2016; de Hevia et al., 2017), supporting the view that these two number-space mappings reflect distinct cognitive phenomena, at least early in life.

Second, a subcomponent of the number-spatial position mapping, which reflects the spontaneous tendency to mentally organize ordered information along a spatially oriented axis, is extended to any ordered dimension even in infancy (Bulf et al., 2017; Bulf et al., 2022). This type of number-spatial location mapping differs from the lateralized one (i.e., left-small/right-large) in that while the first one is related to the ordinality of the information (i.e., first, second, third…), the latter one, as it is also the case for the number-spatial extent mapping, is tied to the information of magnitude (i.e., smaller vs. larger). The fact that numbers can reflect these two properties at the same time, ordinality and cardinality, partially explains why authors often consider that any numerical-spatial effect emanates from a single representational object. However, analogous effects for non-numerical information, such as a SNARC-like effect for items in a grocery list (Previtali et al., 2010), are hardly accounted for by invoking a mental number line.

Finally, the question of an absence or not of a dependency between the number-spatial extent and the number-spatial location mappings has not received, to our knowledge, explicit attention by researchers. However, in one experiment from a larger study investigating the automaticity of the activation of numerical magnitude, Fitousi et al. (2009) observed that the SNARC effect was independent of the SCE in a task where participants had to judge the physical size of Arabic digits, again going against the idea of both number-space mappings reflecting a single construct in adulthood.

**FIGURE 1:**
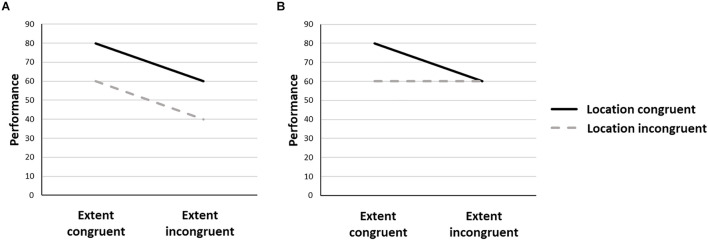
Predicted patterns of performances in the case of distinct **(A)** or single **(B)** representational objects accounting for the number/location and number/spatial extent mappings.

The present study is aimed at further investigating the interaction between the number-spatial extent and the number-spatial location interactions within a single experimental session. If both mappings reflect the same psychological construct, i.e., the same representational object (e.g., large number = large size = right side of space), they should interact with each other. As a consequence, the congruency effect related to one mapping will be modulated by the congruency relative to the other mapping. For instance, if the number/location (resp. number/spatial extent) mapping is congruent, then there will be a number/spatial extent (resp. number/location) mapping, so that congruent trials will lead to higher performance relative to incongruent trials. For number/location (resp. number/spatial extent) incongruent mappings, no difference in performance should be observed between number/spatial extent (resp. number/location) congruent and incongruent trials. However, if both mappings reflect distinct representational objects, the congruency relative to one mapping should not impact the congruency effect related to the other mapping. If we assume that both congruency effects are unaffected by each other, this should in turn, lead to an overall additivity pattern, whereby the highest performance is observed in trials showing a congruency for both mappings, and the lowest performance is observed in trials showing an incongruency for both mappings (Figure 1).

It is possible that the interaction between the two mappings depends on the format of presentation of numbers. According to the infant literature mentioned above, we hypothesize distinct mappings with non-symbolic representations of numbers. However, with education, digits could be mapped onto multiple spatial dimensions (extent and location) in a more holistic manner, leading to stronger interactions between both mappings when processing symbolic numbers.

To test these predictions, we designed a numerical judgment task containing both number-extent and number-location congruent and incongruent trials. In order to directly assess the role of the format of presentation, each participant performed both a non-symbolic and a symbolic version of the task.

## Experiment 1

### Methods

#### Participants

We recruited 77 adult participants using the Prolific online recruitment platform [47 males (2 data missing), mean age = 30.64 years, *SD* = 9.49 (4 data missing)]^1^. They each received a 2.6 euros compensation for their participation. We ran an *a priori* power analysis using G^∗^Power, Version 3.1; Faul et al., 2009 in order to estimate the sample size. The analysis indicated that a sample size of 54 was necessary to detect a medium effect size (Cohen’s *f* = 0.25) in a 2 Format (symbolic, non-symbolic) × 2 Location Congruency (congruent, incongruent) × 2 Size Congruency (congruent, incongruent) repeated measures ANOVA with a power of 0.95 (α = 0.05).

The internal ethical board of the Faculty of Psychology ruled that in light of the potential risks for the participants of the present study, no formal ethical approval by one of the National Ethical Committees was needed in agreement with the Ethical law governing human research in France. Participation was voluntary after obtaining signed informed consent. All participants were tested in accordance with national and international norms governing the use of human research participants.

#### Materials and Procedure

**FIGURE 2:**
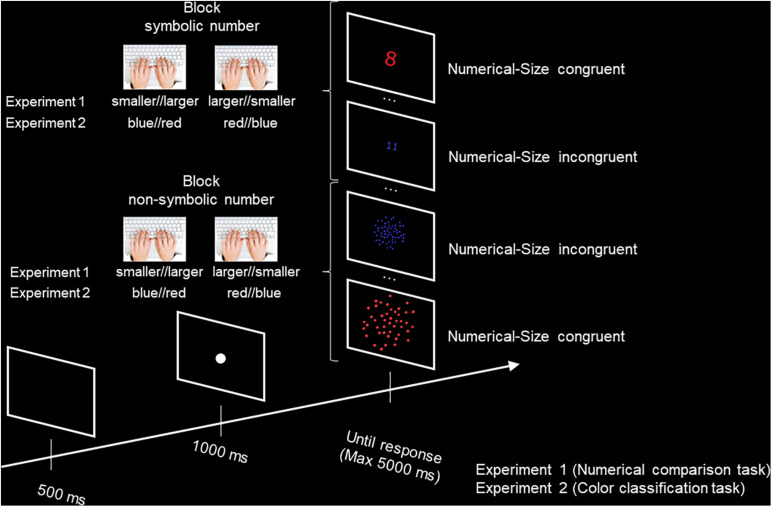
Illustration of the methodology used in Experiment 1 (Numerical comparison task) and Experiment 2 (Color classification task).

The task was programmed using the jsPsych JavaScript library (de Leeuw, 2015), and the data was collected online through the Cognition.run website. The participants were asked to perform symbolic and non-symbolic numerical comparison tasks. In the symbolic comparison task, Arabic digits (1, 2, 3, 4, 5, 7, 8, 9, 10, 11) were presented at the center of the screen and the participants had to decide whether the numbers were smaller or larger than 6, by pressing either the “d” or the “k” key on their computer’s keyboard. In the non-symbolic comparison task, participants had to decide whether the number of dots (6, 7, 8, 9, 10, 40, 44, 48, 52, 56) in centrally presented arrays was smaller or larger than 20, by pressing the “d” or “k” key. In order for the symbolic and non-symbolic tasks to present similar difficulty levels, while avoiding the subitizing range for the non-symbolic task, we used a 1:2 ratio between the reference (20) and the two immediately smaller (10) and larger (40) numbers. This allowed us to use five numbers above the subitizing range (6, 7, 8, 9, 10) and five numbers with matching ratios to 20 (40, 44, 48, 52, and 56). Using the jsPsych calibration plugin, the Arabic digits were set to be presented at a fixed size of either 1 cm × 0.6 cm (small size) or 2 cm × 1.2 cm (large size) at the center of the participants’ screen, while the images of the dot arrays were set to be presented at a fixed size of either 5.8 cm × 5.8 cm (small size) or 11.6 cm × 11.6 cm (large size) at the center of the participants’ screen. The arrays of dots were generated using Matlab. The dots were randomly arranged on the surface of the images, and had a constant size. Thus, the large images showed arrays of dots twice the size of the dots in the small images, and occupying a space twice as large. Half of the stimuli (Arabic digits and dot arrays) were presented in blue over a black background, while the other half was red over a black background. The color and physical size of the stimuli were counterbalanced across both tasks. Each task (symbolic and non-symbolic) consisted of two blocks, one block for each response/key assignment. The order of the tasks was counterbalanced across participants. The order of the response/key assignments (“larger” on the right first, or “larger” on the left first) was constant across both tasks, and counterbalanced across the participants. In both tasks, a trial started with the presentation of a black screen for 500 ms, followed by a central fixation point for 1 s. Then, the stimulus was presented until the participants gave their response. If no response was given, the next trial began after 5 s of stimulus’ presentation (Figure 2). Each task started with eight training trials, during which a feedback on accuracy (“correct” printed in green or “incorrect” printed in red) was given for 1 s, followed by a 500 ms black screen. Each block contained 80 trials, half of which were size-congruent (e.g., large number in large physical size), and the other half were size-incongruent. For each task, one block consisted of location-congruent trials (“small” response on the left, “large” response on the right), while the other block consisted of location-incongruent trials. This led to a total of 320 trials across the two tasks, for a duration of approximately 15 min. The participants were invited to take short breaks if needed between each block and each task.

### Results

The data from 11 participants were removed due to an accuracy rate (AR) below 75% in at least one of the tasks’ blocks. Thus, the reported analyses were performed on a sample of 66 participants. For each participant, we removed the trials with response times either below 150 ms, or larger than three standard deviations above the individual’s average. This led to removing 1.71% of the total number of trials across the analyzed sample. For each participant, we combined ARs and RTs by computing an inverse efficiency score (IES; Townsend and Ashby, 1978, 1983), that is, by dividing participants’ average RTs by their average ARs for each of the eight experimental conditions (2 task × 2 size congruency × 2 location congruency). Note that, in line with the recommendations of Bruyer and Brysbaert (2011), the use of IES was possible due to the high ARs in the tasks (mean = 0.96, *SD* = 0.04 in Experiment 1, mean = 0.96, *SD* = 0.04 in Experiment 2), and the absence of speed-accuracy trade-offs [*r*(65) = 0.1, *p* = 0.45 in Experiment 1, *r*(67) = 0.05, *p* = 0.68 in Experiment 2].

**FIGURE 3:**
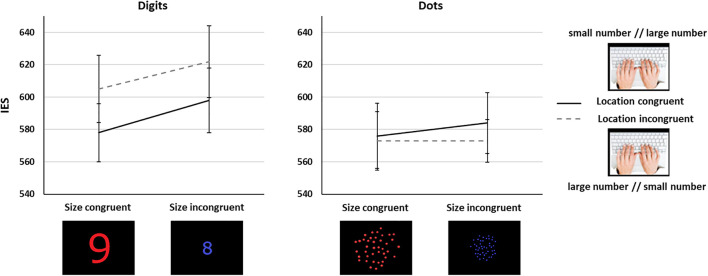
Interaction between number/location and number/spatial extent mappings in Experiment 1 for the symbolic (left) and the non-symbolic (right) tasks. Error bars show standard error of the mean (SEM).

We ran a 2 Task (symbolic, non-symbolic) × 2 Size Congruency (size congruent, size incongruent) × 2 Location Congruency (location congruent, location incongruent) repeated measures ANOVA on the IES. We observed a main effect of Task [*F*(1,65) = 4.89, *p* = 0.03, η^2^*_*p*_* = 0.07], with lower performance in the symbolic (*IES* = 601, *SD* = 165) than in the non-symbolic task (*IES* = 577, *SD* = 143). The results also showed a classic main effect of Size Congruency [*F*(1,65) = 17.21, *p* < 0.001, η^2^*_*p*_* = 0.21], with lower performance in the size incongruent (*IES* = 594, *SD* = 153) than in the size congruent (*IES* = 583, *SD* = 156) trials. There was a significant Task × Location Congruency interaction [*F*(1,65) = 7.41, *p* = 0.008, η^2^*_*p*_* = 0.1]. *Post hoc* Bonferroni corrected comparisons indicated a trend toward an effect of Location congruency (*p* = 0.19) in the symbolic, but not in the non-symbolic task (*p* = 1). Critically, there was a significant difference between the performance in location incongruent trials between the non-symbolic and the symbolic tasks (*p* = 0.01), but not in location congruent trials (*p* = 1), showing that location incongruency significantly worsened performance with Arabic digits but not with dot arrays. There were no other significant effects. In particular, we did not observe a significant interaction involving Size and Location Congruency (*Fs* < 1.07, *ps* > 0.31, Figure 3), in line with the prediction in Figure 1A. We verified that the same analyses run on RTs yielded similar results, with a significant main effect of Size Congruency [*F*(1,65) = 15.98, *p* < 0.001], and a significant Task × Location Congruency interaction [*F*(1,65) = 5.40, *p* = 0.02]. We only found a significant main effect of Size Congruency when analyzing ARs [*F*(1,65) = 6.318, *p* = 0.014], which could possibly be due to ceiling effects.

### Interim Discussion

We found an effect of congruency between the numerical magnitude and the physical magnitude, for both symbolic and non-symbolic stimuli, so that congruent trials (i.e., when larger numerical quantities were larger in size, and smaller numerical quantities were smaller in size) had a higher performance than incongruent ones (i.e., when numerical quantities and their sizes differed, one being small and the other large). The overall lower performance with digits than with non-symbolic numbers could be due to the ratio differences, which were more pronounced for non-symbolic stimuli, and therefore comparison was easier and faster for this numerical format.

We observed a number/location congruency effect (SNARC) only for digits. While performance for dots was not affected by the location of the response side (no cost nor advantage for location-incongruent and location-congruent trials, respectively), and performance was similar to that for location-congruent trials in digits, there was a significant performance cost for digit trials, compared to dot trials, in location-incongruent trials. Therefore, the spatial location of the response button affected symbolic but not non-symbolic numerical comparisons. Studies investigating non-symbolic SNARC effects are scarcer than in the symbolic domain, with mixed results (Cleland et al., 2020). However, a few studies in adults have reported SNARC or SNARC-like effects with non-symbolic numbers, especially when numerical magnitude was relevant to the task (Zhou et al., 2016; Nemeh et al., 2018), in contradiction with our results. The heightened importance of spatial location of response buttons for Arabic digits might derive from the fact that adults have abundant exposure to digits arranged on a horizontal (left-right oriented) space, while this spatial layout is rarely, if ever, observed with non-symbolic numerosities. Finally, there was no interaction between the two types of number-spatial mappings, suggesting that both mappings act independently.

Quantity might have a more relevant role for the SCE, while the SNARC effect might be boosted in a task highlighting order. Since we used an explicit numerical comparison task, it is possible that the number-spatial extent mapping was enhanced, in detriment of the number-spatial position mapping, and might account for the absence of location congruency effects for non-symbolic numbers. These results are in line with Fitousi et al.’s (2009) study, in which the size judgment task was also emphasizing the number-spatial extent mapping. Additionally, in our task, the number/spatial extent and number/location congruencies varied at different levels. While spatial extent congruency was manipulated at the trial level, location congruency varied between blocks (based on the response-key assignment). Since we aimed at keeping the task instructions identical across the two numerical formats, we ran the same experiment, except that we engaged participants in implicit non-symbolic and symbolic numerical tasks, by asking them to judge the color of the numerical stimuli. In this way, we avoided favoring a mental representation in terms of quantity or order, and congruencies relative to spatial extent and location both varied at the trial level.

## Experiment 2

### Methods

#### Participants

We recruited 73 new adult participants using the Prolific online recruitment platform (see text footnote 1; 58 males, mean age = 26.56 years, *SD* = 6.92). They each received a 2.6 euros compensation for their participation.

The internal ethical board of the Faculty of Psychology ruled that in light of the potential risks for the participants of the present study, no formal ethical approval by one of the National Ethical Committees was needed in agreement with the Ethical law governing human research in France. Participation was voluntary after obtaining signed informed consent. All participants were tested in accordance with national and international norms governing the use of human research participants.

#### Materials and Procedure

The materials and procedure were the same as in Experiment 1, except for the instructions. The participants performed a symbolic and a non-symbolic task, in which they were instructed to decide whether the presented stimulus was blue or red, by pressing one of the two keys on their keyboard. Given these new instructions, the location-congruency of the trials was now counterbalanced within each block. For instance, if the response/key assignment was “red on the left and blue on the right,” half of the large numbers (the blue ones) were responded to with the right-hand key (location-congruent), while the other half (the red ones) were responded to with the left-hand key (location-incongruent).

### Results

As in Experiment 1, we removed the data from five participants whose ARs were below 75% in at least one of the experimental blocks, leading to an analyzed sample of 68 participants^2^. 1.98% of the entire dataset was removed due to RTs faster than 150 ms or slower than 3 standard deviations above the individual’s average. We ran a 2 Task (symbolic, non-symbolic) × 2 Size Congruency (size congruent, size incongruent) × 2 Location Congruency (location congruent, location incongruent) repeated measures ANOVA on the IES.

**FIGURE 4:**
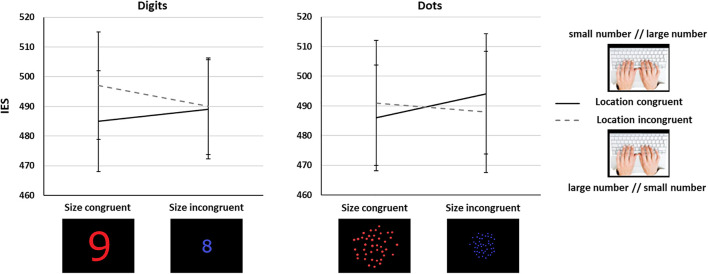
Interaction between number/location and number/spatial extent mappings in Experiment 2 for the symbolic (left) and the non-symbolic (right) tasks. Error bars show standard error of the mean (SEM).

We did not observe main effects of Task nor Size Congruency (*Fs* < 1). There was only a trend toward a main effect of Location Congruency [*F*(1,67) = 2.38, *p* = 0.13]. None of the interactions involving Task reached significance (*Fs* < 1.59, *ps* > 0.21). Importantly, and contrary to what was observed in Experiment 1, we found a significant interaction between Size and Location Congruency [*F*(1,67) = 6.52, *p* = 0.01, η^2^*_*p*_* = 0.09, Figure 4]. *Post hoc* Bonferroni corrected comparisons revealed a significant Location Congruency effect only for size congruent trials (location incongruent: *IES* = 494, *SD* = 161, location congruent: *IES* = 485, *SD* = 143, *t* = 2.92, *p* = 0.03), but not for size incongruent trials (location incongruent: *IES* = 489, *SD* = 151, location congruent: *IES* = 491, *SD* = 152, *t* = 0.8, *p* = 1), in line with the prediction in Figure 1B. Regarding Size congruency, although performance was lower for size incongruent than size congruent trials only in the location congruent trials, this difference did not reach statistical significance (*p* = 0.36; all other contrasts were not significant, all *p*s > 0.6). As in Experiment 1, we observed similar results with only a significant Size × Location Congruency interaction [*F*(1,67) = 4.48, *p* = 0.04] when using RTs, and no significant effect when using ARs.

### Interim Discussion

When using an implicit task, i.e., a color judgment task, participants did not show anymore a main effect of size congruency, possibly because this effect was weakened when avoiding explicit magnitude coding. However, and in contrast to Experiment 1, we found a location congruency effect only in size congruent trials, so that there was an advantage in performance for smaller quantities responded on the left and larger quantities on the right when the size of stimuli aligned with their numerical magnitude. This effect was present irrespective of the numerical format, involving both symbolic and non-symbolic trials.

## General Discussion

In this study we aimed at directly investigating the question of whether there is an interaction between two number-space mappings, i.e., the number/spatial extent and the number/spatial location mappings. We reported that, when using a numerical magnitude judgment task, the two mappings did not interact: the difference in performance between congruent and incongruent trials relative to one mapping was the same for congruent and incongruent trials relative to the other mapping (in line with the prediction Figure 1A). However, in the context of an implicit task, i.e., a color judgment task, using the same stimuli, the two mappings interacted, with stronger congruency effect relative to one mapping when the trials were congruent relative to the other mapping (in line with the prediction Figure 1B).

Altogether, our results suggest that both the number/extent and the number/location mappings can tap onto a shared representational object, but that its activation critically depends on the experimental context, and can manifest itself in different ways. Several factors could be at play. First, the reliance on a shared representational object for both mappings could depend on whether magnitude is implicitly or explicitly activated by the task. Both in Fitousi et al. (2009) and in the current study, using a task whereby magnitude (either numerical or non-numerical) was explicitly activated yielded no interaction between the two mappings. As mentioned previously, it is possible that the interaction between both mappings depends on how much the experimental context emphasizes one mapping over the other. By having instructions based on numerical magnitude or physical size, the number/spatial extent mapping might be more activated, preventing the use of a shared representational object. On a related note, previous studies have found that the nature of the number/location mappings, reflected by the SNARC effect, could differ depending on the task (Gevers et al., 2006), with a more categorical association observed in the context of a numerical magnitude judgment task, and a more continuous association in the context of a parity judgment task. It is possible that the two mappings only interact in an experimental context typically eliciting a more continuous number/location mapping.

Second, for an interaction to be observed between the two mappings, another factor could be the level at which the different types of information are being manipulated. When using a magnitude-relevant task, the number/location mapping can only be analyzed by comparing different blocks of trials, corresponding to the different response-key assignments (e.g., “more on the right” vs. “more on the left”). Using a task whereby numerical magnitude is irrelevant, such as in Experiment 2, allows us to analyze the number/location mapping across trials (similarly to the analysis of the number/extent mapping), since the numerical magnitude is independent of the response side. This methodological factor could also contribute to balancing out the weight of each mapping, yielding to their interaction, as observed in Experiment 2. Further studies will be needed to investigate the exact contextual conditions that elicit an interaction between the number/extent and the number/location mappings.

Our results did not show any evidence for an effect of format on the interaction between the two number-space mappings. While Experiment 1 showed an effect of the format of presentation (symbolic vs. non-symbolic) on the number-location mapping, in both experiments the interaction between the two mappings did not differ significantly depending on the format. This could indicate a continuity in the number-space associations between non-symbolic and symbolic representations of magnitude. Developmental studies would help to shed more light on this question.

Altogether, our results support the idea of the existence of a shared spatial representational object with a directionality (from left-to-right in our group of participants), and on which smaller numbers are associated with smaller spatial extents, and larger numbers with larger spatial extents (de Hevia, 2021). In this regard, the mental number line could account both for the number/location and the number/extent mappings. However, and in line with previous studies investigating the SNARC effect in particular (Viarouge et al., 2014), we see that this shared representation can be activated flexibly depending on the experimental context. Thus, while we provide evidence for the existence of a shared representation, our data suggests that its activation is not automatic in any task using numerical stimuli.

## Data Availability Statement

The raw data supporting the conclusions of this article will be made available by the authors upon request, without undue reservation.

## Ethics Statement

Ethical review and approval was not required for the study on human participants in accordance with the local legislation and institutional requirements. The patients/participants provided their written informed consent to participate in this study.

## Author Contributions

AV and MH equally contributed to the conception and design of the study, wrote the first draft of the manuscript, revised it, and approved the submitted version. AV performed the statistical analysis. Both authors contributed to the article and approved the submitted version.

## Conflict of Interest

The authors declare that the research was conducted in the absence of any commercial or financial relationships that could be construed as a potential conflict of interest.

## Publisher’s Note

All claims expressed in this article are solely those of the authors and do not necessarily represent those of their affiliated organizations, or those of the publisher, the editors and the reviewers. Any product that may be evaluated in this article, or claim that may be made by its manufacturer, is not guaranteed or endorsed by the publisher.

## Footnotes

1
www.prolific.co

2 Although power analysis indicated a sample size of 54, no sequential testing was performed and data were only analyzed once all the data were collected (*N* = 77 in Experiment 1, *N* = 73 in Experiment 2). In order to test for the robustness of the interaction in Experiment 2 vs. the absence of interaction in Experiment 1, we ran 100 analyses on random sub-samples of 54 participants. The significant Location × Size Congruency interaction reported in Experiment 2 was observed 74 out of 100 times (*p* < 0.001, binomial test), while it was significant only 1 out of 100 times in Experiment 1. Moreover, the significant Task × Location Congruency interaction reported in Experiment 1 was observed 87 out of 100 times (*p* < 0.001, binomial test).
